# Prevalence of Dental Caries and Its Association With Oral Hygiene Practices Among Primary School Children Aged 6-11 Years in North India: A Cross-Sectional Study

**DOI:** 10.7759/cureus.106832

**Published:** 2026-04-11

**Authors:** Mrinal Kalhuria, Abhishek Dhindsa, Sulekha Manhas, Shagun Kaushik, Reena Rani, Saurav Dutta, Neetu Jain, Seema Gupta

**Affiliations:** 1 Department of Paediatric and Preventive Dentistry, Maharishi Markandeshwar College of Dental Sciences and Research, Ambala, IND; 2 Department of Dentistry, Adesh Medical College and Hospital, Mohri, IND; 3 Department of Paediatric and Preventive Dentistry, Genesis Institute of Dental Sciences and Research, Ferozepur, IND; 4 Department of Dentistry, Smile Dental Clinic, Panchkula, IND; 5 Department of Paediatric and Preventive Dentistry, Santosh Dental College and Hospital, Ghaziabad, IND; 6 Department of Prosthodontics and Crown and Bridge, National Dental College, Derabassi, IND; 7 Department of Orthodontics, Kothiwal Dental College and Research Centre, Moradabad, IND

**Keywords:** children, dental caries, oral hygiene, practice, prevalence, school

## Abstract

Introduction: Dental caries is one of the most prevalent chronic diseases affecting children worldwide and remains a major public health concern. It is a multifactorial condition influenced by oral hygiene practices, dietary habits, and behavioral factors. The mixed dentition period (6-11 years) is particularly critical as children are more susceptible to developing caries due to changing oral environments and inconsistent oral hygiene practices. The aim of the present study was to assess the prevalence of dental caries and its association with oral hygiene practices among primary school children aged 6-11 years in the district of Panchkula, Haryana, India.

Materials and methods: A cross-sectional study was conducted among 1040 school-going children selected using multistage random sampling. Dental caries in the permanent dentition was recorded using the decayed-missing-filled teeth (DMFT) index, whereas caries in the primary dentition was assessed using the decayed-extracted-filled teeth (deft) index. Oral hygiene practice was evaluated using a structured questionnaire. Data were analyzed using appropriate statistical tests, including chi-square analysis, with the significance level set at p < 0.05.

Results: The overall prevalence of dental caries was 84.4%. No significant association was found between sex and the prevalence of caries (p > 0.05). However, the prevalence of caries increased significantly with age (p < 0.001). Oral hygiene practices showed a statistically significant association with dental caries (p < 0.0001), with a higher prevalence among children with poor brushing habits, infrequent brushing, improper cleaning methods, high consumption of sweets, and irregular dental visits. Mean DMFT and deft scores increased significantly with age (p = 0.001). Additionally, children with poor oral hygiene scores demonstrated significantly higher DMFT and deft values than those with good and fair hygiene (p < 0.001).

Conclusion: This study revealed a high prevalence of dental caries among children in the mixed dentition stage, with oral hygiene practices playing a significant role. Early preventive interventions, improved oral health education, and parental involvement are essential for reducing the burden of dental caries in children.

## Introduction

Dental caries is one of the most prevalent chronic diseases worldwide and significantly affects oral health and overall well-being. According to the World Health Organization (WHO), untreated dental caries affects billions of people globally, with high burdens in both permanent and primary dentition among children [1]. It impairs eating, speech, and social interactions while contributing to pain, nutritional deficiencies, growth issues, and reduced quality of life, particularly in developing nations, where rising sugar consumption, limited preventive care, and inadequate oral hygiene exacerbate the problem [2].

In children, dental caries is a multifactorial disease influenced by bacterial activity, host susceptibility, cariogenic diet, time, and poor oral hygiene practices [3]. Globally, 60-90% of school-aged children experience caries, with greater severity in low- and middle-income countries. Recent data indicate persistently high rates, including increasing trends in early childhood caries, over the past decade [1,4]. In India, dental caries poses a major public health challenge, with meta-analyses reporting an overall prevalence of approximately 54% and age-specific rates of approximately 52% in children aged 3-18 years [5]. Studies among schoolchildren often show higher prevalence in various regions, with higher burdens in primary (deciduous) teeth due to improper hygiene, frequent sugary intake, and socioeconomic barriers [6]. Variations occur by geography, urban-rural divide, socioeconomic status, parental education, and access to oral health resources, underscoring the need for localized epidemiological data to guide interventions [7].

The district of Panchkula, Haryana, India, represents a region with evolving oral health patterns influenced by mixed urban-rural demographics and changing lifestyles. Limited specific data exist for primary school children, highlighting the importance of targeted assessment. The aim of the present study was to assess the prevalence of dental caries and its association with oral hygiene practices among primary school children aged 6-11 years in the district of Panchkula. The objectives were (1) to determine the overall prevalence of dental caries using the decayed-missing-filled teeth (DMFT) index for permanent dentition and decayed-extracted-filled teeth (deft) index for primary dentition; (2) to evaluate oral hygiene practices, including frequency, method, timing, and supervision of tooth cleaning; and (3) to analyze the statistical association between these practices and caries prevalence in this population.

## Materials and methods

The present study was a cross-sectional epidemiological survey conducted at Swami Devi Dyal Hospital and Dental College, Panchkula, India, from April 2022 to September 2022. Ethical clearance was obtained from the Institutional Ethical Committee of the College (SDDHDC/IEC/Mar2022/024, dated March 15, 2022) prior to commencement. Informed consent was obtained from the school authorities and parents/guardians of the participating children to ensure voluntary participation and the right to withdraw at any time.

A multi-stage random sampling technique was employed to select study participants. In the first stage, government primary schools in the district of Panchkula were identified and listed. A total of 62 government primary schools were identified in the district of Panchkula, out of which 10 schools were randomly selected using a multistage sampling technique for inclusion in the study. A random selection of schools was made proportionate to their distribution across urban and rural areas within the district. In the second stage, eligible children aged 6-11 years (corresponding to primary classes) were randomly selected from the attendance registers of the chosen schools, aiming for an approximately equal representation across age groups and sexes, where possible. The final sample comprised 1,040 school-going children from the selected primary government schools.

The sample size for the present study was estimated using G*Power (version 3.2.9; Heinrich-Heine-Universität Düsseldorf, Düsseldorf, Germany). Considering a statistical power of 80% and an alpha error of 5%, the minimum required sample size was calculated to detect a significant association (effect size of 0.4) in dental caries prevalence and the age groups. Based on the medium effect size of 0.4 and the selected statistical tests (correlation analysis), the estimated minimum sample size required was 886 participants [2]. However, to improve the precision and reliability of the findings, 1040 children were included in the final analysis at higher power.

Data collection involved both a structured questionnaire about basic demographic details (Appendix A), oral hygiene practices (Appendix B), and a clinical oral examination (Appendix C). A pre-tested, structured questionnaire was administered to gather information on oral hygiene practices, including whether the child cleaned their teeth (yes/no), who performed the cleaning (self, parents, grandparents, or others), the method used (toothbrush, finger, datun/manjan/coal ash, or others), frequency of cleaning per day (once, twice, three times, or others), and timing of cleaning (before breakfast only, before breakfast and after dinner, or other combinations), presented in a bilingual (English/Hindi) format with age-appropriate language. The questionnaire was completed with the assistance of teachers or parents, when necessary, to ensure accuracy among younger children.

Content validity was confirmed by a panel of five experts (pediatric dentists, public health dentist, epidemiologist, supervisor), achieving an Item Content Validity Index (I-CVI) ≥ 0.78 and a Scale Content Validity Index (S-CVI/Ave) > 0.90, after minor wording refinements. Face validity and operational feasibility were evaluated through a pilot study with 25 children and their parents and teachers, confirming easy comprehension (average completion time 5-7 min) and no major ambiguities. Reliability testing showed excellent test-retest agreement (Cohen’s kappa 0.82-0.95 over 7-10 days) and acceptable internal consistency (Cronbach’s alpha = 0.81). A second pre-test with 20 additional children verified readiness for field use, ensuring that the tool reliably captured accurate self-reported data during face-to-face administration with teacher assistance in the main study.

Clinical examinations were scheduled on specific dates during the zero period (early morning school hours) to minimize disruption. Prior to the examination, each participating child was provided with a new junior toothbrush and toothpaste and instructed to brush their teeth under supervision. This step standardized plaque removal and facilitated accurate detection of carious lesions. Intraoral examinations were conducted in a well-lit room (natural daylight supplemented by artificial light, if needed) with the child seated comfortably on a regular chair. The principal investigator, calibrated with the supervisor and co-supervisor for consistency and reproducibility, performed all examinations using standard infection control measures, including gloves, masks, and sterilization of the instruments.

Dental caries were recorded separately for permanent and primary dentition using the decayed-missing-filled teeth (DMFT) index for permanent teeth and the decayed-extracted-filled teeth (deft) index for primary dentition [8]. According to these guidelines, a tooth was classified as decayed if it presented with a detectable cavity with a softened floor, undermined enamel, or softened wall. Lesions without cavitation were considered sound. Missing teeth were recorded only if they were extracted due to caries (not for orthodontic or other reasons), and filled teeth included those with restorations. For primary dentition, the deft index adopts similar principles, recording decayed, extracted (due to caries), and filled primary teeth. No radiographs were used, as the study relied on a clinical visual-tactile examination with a plain mouth mirror and probe.

All the recorded data were tabulated, coded, and entered into a spreadsheet for statistical analysis. Data were analyzed using the statistical software IBM SPSS Statistics for Windows, version 26 (IBM Corp., Armonk, NY, USA). The normality of the data distribution was assessed and confirmed using the Shapiro-Wilk test. Categorical variables were expressed as frequencies and percentages, whereas continuous variables were presented as means and standard deviations. The association between the prevalence of dental caries and variables, such as age and sex, was evaluated using the chi-square test. Similarly, the relationship between oral hygiene practices and caries prevalence was assessed using the chi-squared test. Differences in DMFT and oral hygiene scores among the age groups were analyzed using one-way analysis of variance (ANOVA), followed by post-hoc analysis for pairwise comparisons. Statistical significance was set at p < 0.05.

## Results

A total of 1040 children were included in the analysis, representing a balanced distribution across age groups and a predominance of female participants. The overall prevalence of dental caries in the study population was 84.4%, indicating a high burden of dental caries among children in the mixed dentition stage. The study population also reflected varied parental educational backgrounds, indicating a heterogeneous sample (Table 1).

**Table 1:** Demographic characteristics of the study population. Data are presented as frequency (n) and percentage (%).

Characteristic	Category	Number n (%)
Age group (years)	6–7	307 (29.5)
	8–9	383 (36.8)
	10–11	350 (33.7)
Sex	Male	354 (34.0)
	Female	686 (66.0)
Parent/guardian education	No formal	156 (15.0)
	Primary	260 (25.0)
	Secondary	416 (40.0)
	Graduate or above	208 (20.0)

No statistically significant association was observed between sex and dental caries prevalence across any age group, suggesting that caries occurrence was comparable between males and females (Table 2).

**Table 2:** Prevalence of dental caries by sex across groups. Chi-square test used to assess association between sex and caries prevalence, p < 0.05 considered statistically significant. Data are presented as frequency (n) and percentage (%).

Age group (years)	Sex	Total (n)	Caries n (%)	χ² value	p-value
6–7	Male	104	78 (75.0)	0.001	0.98
	Female	203	152 (74.9)		
8–9	Male	130	111 (85.4)	0.015	0.90
	Female	253	215 (85.0)		
10–11	Male	120	111 (92.5)	0.062	0.80
	Female	230	211 (91.7)		
Overall	Male	354	300 (84.7)	0.047	0.83
	Female	686	578 (84.3)		

By contrast, age demonstrated a strong and statistically significant relationship with dental caries. An increasing trend in caries prevalence was observed with advancing age, indicating a higher disease burden among older children (Table 3).

**Table 3:** Association of prevalence of dental caries by age groups with Chi-square test. Data are presented as frequency (n) and percentage (%), *p < 0.05 considered statistically significant.

Age group (years)	Total (n)	Caries n (%)	χ² value	p-value
6–7	307 (29.5)	230 (74.9)	39.04	< 0.001*
8-9	383 (36.8)	326 (85.1)		
10–11	350 (33.7)	322 (92.0)		

Oral hygiene practices were significantly associated with caries prevalence. Children with inadequate oral hygiene behaviors, including infrequent brushing, use of alternative cleaning methods, irregular rinsing habits, frequent consumption of sweets, and lack of regular dental visits, exhibited a higher prevalence of dental caries. Conversely, healthier practices were associated with a lower occurrence of caries (Table 4).

**Table 4:** Association between oral hygiene practices and dental caries prevalence with Chi-square test. Data are presented as frequency (n) and percentage (%), *p < 0.05 considered statistically significant.

Oral hygiene practice	Response	Caries present (n = 878)	Caries absent (n = 162)	χ² value	p-value
Daily brushing	Yes	702 (82.0%)	154 (18.0%)	21.44	< 0.001*
	No	176 (95.7%)	8 (4.3%)		
Brushing frequency	Once	527 (91.5%)	49 (8.5%)	77.85	< 0.001*
	Twice	307 (79.1%)	81 (20.9%)		
	> Twice	44 (57.9%)	32 (42.1%)		
Toothbrush use	Toothbrush + paste	615 (80.8%)	146 (19.2%)	27.56	< 0.001*
	Other (powder/finger)	263 (94.3%)	16 (5.7%)		
Rinse after meals	Always	176 (73.0%)	65 (27.0%)	39.96	< 0.001*
	Sometimes	439 (85.7%)	73 (14.3%)		
	Never	263 (91.6%)	24 (8.4%)		
Sweets frequency	Daily	351 (93.6%)	24 (6.4%)	90.24	< 0.001*
	Weekly	263 (86.5%)	41 (13.5%)		
	Occasionally	176 (75.5%)	57 (24.5%)		
	Rarely	88 (68.8%)	40 (31.2%)		
Dental visit pattern	Regular	44 (64.7%)	24 (35.3%)	74.74	< 0.001*
	Only when problem	439 (81.9%)	97 (18.1%)		
	Never	395 (90.6%)	41 (9.4%)		

A similar pattern was evident when oral hygiene was assessed using composite scores, wherein multiple behavioral parameters were aggregated into a single index. Children categorized with poor composite scores showed a markedly higher prevalence of dental caries compared to those with fair and good scores, highlighting the cumulative effect of oral hygiene practices on caries risk (Table 5).

**Table 5:** Association between oral hygiene practice score categories and caries prevalence with Chi-square test. Data are presented as frequency (n) and percentage (%), *p < 0.05 considered statistically significant.

Caries status	Good (score 9–12)	Fair (score 5–8)	Poor (score 0–4)	χ² value	p-value
Present (n = 878)	108 (12.3)	408 (46.5)	362 (41.2)	17.81	< 0.001*
Absent (n = 162)	71 (43.8)	72 (44.4)	19 (11.8)		

The analysis of DMFT/deft scores further supported these findings. A progressive increase in the mean DMFT/deft values was observed with age, with statistically significant differences between all age groups, reflecting cumulative caries experience over time (Table 6).

**Table 6:** Mean DMFT/deft scores by age groups. One-way analysis of variance (ANOVA) followed by post-hoc (Tukey) test, *p < 0.05 considered statistically significant. DMFT: decayed-missing-filled teeth, deft: decayed-extracted-filled teeth.

Parameters	Age group (years)	N (%)	Mean ± SD	F	p	6–7 vs 8–9	6–7 vs 10–11	8–9 vs 10–11
						p (t value)	p (t value)	p (t value)
DMFT	6-7	307 (29.5)	2.19 ± 1.70	163.82	< 0.001*	< 0.001* (9.35)	< 0.001* (18.09)	< 0.001* (9.44)
	8-9	383 (36.8)	3.77 ± 2.16					
	10-11	350 (33.7)	5.31 ± 2.61					
deft	6-7	307 (29.5)	1.23 ± 1.64	317.5	< 0.001*	< 0.001* (13.10)	< 0.001* (25.19)	< 0.001* (13.07)
	8-9	383 (36.8)	3.11 ± 1.89					
	10-11	350 (33.7)	4.92 ± 2.04					

Additionally, the mean DMFT/deft scores varied significantly according to oral hygiene status. Children with poor hygiene scores exhibited substantially higher DMFT/deft values than those with fair or good hygiene, reinforcing the strong association between oral hygiene practices and caries severity (Table 7).

**Table 7:** Mean DMFT/deft by oral hygiene practice score categories. One-way ANOVA followed by post-hoc (Tukey) test, *p < 0.05 considered statistically significant. DMFT: decayed-missing-filled teeth, deft: decayed-extracted-filled teeth.

Parameters	Oral hygiene score category	N (%)	Mean ± SD	F	p	Good vs. fair	Good vs. poor	Fair vs. poor
						p (t value)	p (t value)	p (t value)
DMFT	Good (9–12)	108 (12.3)	1.82 ± 1.91	118.1	< 0.001*	< 0.001* (23.4)	< 0.001* (12.7)	< 0.001* (21.4)
	Fair (5–8)	408 (46.5)	3.51 ± 2.52					
	Poor (0–4)	362 (41.2)	5.22 ± 2.81					
deft	Good (9–12)	108 (12.3)	0.91 ± 1.23	194.7	< 0.001*	< 0.001* (13.82)	< 0.001* (22.38)	< 0.001* (12.94)
	Fair (5–8)	408 (46.5)	2.89 ± 1.63					
	Poor (0–4)	362 (41.2)	4.92 ± 2.56					

## Discussion

The present study provides important insights into the epidemiology of dental caries among children and highlights the multifactorial nature of the disease, particularly the role of behavioral and age-related factors. One of the key observations was the absence of a significant association between sex and the prevalence of caries. This finding aligns with several epidemiological studies that suggest that dental caries is not inherently influenced by biological sex in early childhood, but rather by shared environmental exposures and behavioral patterns [9,10]. Similarly, the study by Saravanan et al. reported no sex-based differences in caries prevalence, reinforcing the notion that preventive strategies should be uniformly targeted rather than sex-specific [11].

In contrast, age has emerged as a strong determinant of caries experience. The progressive increase in the prevalence and severity of caries with advancing age reflects the cumulative nature of the disease. Prolonged exposure to cariogenic challenges combined with inadequate oral hygiene practices over time contributes to this trend. Comparable findings have been reported in studies by Saravanan et al. and Kassebaum et al., where older children exhibited a significantly higher caries burden [11,12]. This emphasizes the need for early preventive interventions before irreversible disease patterns are established. The overall prevalence of caries observed in the present study was notably high, indicating a substantial burden of disease among children in the mixed dentition stage. Similar high prevalence rates have been reported in previous studies, highlighting that dental caries remains a significant public health concern in pediatric populations [1,4,12].

Oral hygiene practices were significantly associated with caries occurrence, underscoring their critical role in disease prevention. Children with inadequate brushing frequency, improper cleaning methods, and poor rinsing habits are more likely to develop caries. These findings are consistent with those of previous studies that highlighted oral hygiene behavior as a primary modifiable risk factor for dental caries [13,14]. Additionally, the use of non-conventional cleaning methods, such as finger or tooth powder, is associated with poorer outcomes, likely due to ineffective plaque removal [15].

Dietary habits, particularly the frequency of sugar consumption, also showed a strong relationship with caries prevalence. Frequent intake of sweets increases the availability of fermentable carbohydrates, promoting acid production by oral bacteria and subsequent enamel demineralization. This observation is in agreement with the classic Keyes triad and is supported by studies such as Moynihan and Kelly, which established a clear link between sugar consumption and dental caries [16]. The high prevalence of caries among children with daily sweet food consumption highlights the urgent need for dietary counseling as part of a preventive program.

Dental service utilization patterns further influenced the caries experience. Children who visited the dentist only when symptomatic or never had a higher prevalence of caries than those who attended regular check-ups. This finding reflects a common trend in developing countries where dental care is largely treatment-oriented rather than preventive. Similar observations were reported by Watt et al., emphasizing the importance of shifting from a curative to a preventive model of oral healthcare [17].

The significant association between oral hygiene scores and caries status provides additional evidence to support the cumulative impact of daily oral care practices. Children with poorer oral hygiene scores exhibited a higher disease burden, indicating that composite indices can effectively capture behavioral risk factors. Furthermore, the analysis of the mean DMFT scores across age groups and hygiene categories reinforces the progressive and preventable nature of dental caries. These findings are in line with those reported by Featherstone, who described dental caries as a dynamic process influenced by the balance between demineralization and remineralization [18].

Limitations

Despite its strengths, the present study had certain limitations. The cross-sectional design limits the ability to establish causal relationships between the risk factors and dental caries. Self-reported oral hygiene practices may be subject to recall or social desirability biases, potentially affecting accuracy. Additionally, factors such as fluoride exposure, socioeconomic status beyond parental education, and dietary patterns have not been comprehensively assessed. The study was geographically restricted, which may limit the generalizability of the findings to broader populations.

Implications and recommendations for society

This study’s findings have important implications for public health. There is a clear need to strengthen oral health education programs targeting both children and parents, with an emphasis on proper brushing techniques, frequency, and the use of appropriate cleaning aids. School-based preventive programs, including supervised tooth brushing and fluoride application, may play a crucial role in reducing the burden of caries. Dietary counseling should be integrated into routine healthcare, focusing on reducing the frequency of sugar intake rather than only the quantity. Public health policies should also promote regular dental checkups through awareness campaigns and improve accessibility to preventive dental services. Furthermore, the integration of oral health into primary healthcare systems can enhance the early detection and management of dental caries. Community-level interventions, including the training of healthcare workers and the involvement of schools, can significantly improve oral health outcomes in children.

## Conclusions

The present study highlights the high burden of dental caries among children in the mixed dentition period, with age and oral hygiene practices emerging as significant determinants. While sex showed no influence, increasing age and poor oral hygiene behaviors were strongly associated with greater caries experience. Unfavorable dietary habits and irregular dental visits further contributed to disease prevalence. These findings emphasize that dental caries are largely preventable through early intervention, improved oral hygiene practices, and behavioral modifications. Strengthening school-based oral health programs, promoting regular dental check-ups, and enhancing parental awareness are essential steps toward reducing the burden of caries in children.

## Appendices

Appendix A 

**Table 8:** Section A - Patient information sheet.

Prevalence of Dental Caries and its Association with Oral Hygiene Practices among Primary School Children (Questionnaire)			
Identification Details			
Variable			Information
School Name			__________________________
School Type			☐ Government ☐ Private
Student ID			__________________________
Date of Examination			____ / ____ / ______
Examiner Name			__________________________
Section A: Demographic Details			
Question			Response
Age (years)			______
Gender			☐ Male ☐ Female
Class/Grade			☐ I ☐ II ☐ III ☐ IV ☐ V
Parent/Guardian Education			☐ No formal education ☐ Primary ☐ Secondary ☐ Graduate or above
Parent/Guardian Occupation			__________________

Appendix B 

**Table 9:** Section B - Oral hygiene practice information. Oral Hygiene Practice Score - Good: 9 – 15, Fair: 5 – 8, Poor: 0 – 4.

Section B: Oral Hygiene Practices			
Question	Response		Maximum score
1. Do you brush your teeth daily?	☐ Yes ☐ No		1
2. Frequency of brushing	☐ Once ☐ Twice ☐ > Twice		2
3. What do you use to brush?	☐ Toothbrush + toothpaste ☐ Tooth powder ☐ Finger		2
4. Do you rinse mouth after meals?	☐ Always ☐ Sometimes ☐ Never		2
5. Frequency of eating sweets/chocolates	☐ Daily ☐ Weekly ☐ Occasionally ☐ Rarely		3
6. Do you clean your tongue while brushing?	☐ Yes ☐ No		1
7. How often do you change your toothbrush?	☐ 3 months ☐ 6 months ☐ Only when damaged		2
8. Dental visit pattern	☐ Regular ☐ Only when problem ☐ Never		2

Appendix C

**Table 10:** Section C - Information about dental caries. DMFT: decayed-missing-filled teeth, deft: decayed-extracted-filled teeth.

Section C: Clinical Examination (Dental Caries)	
Primary Dentition (deft)	
Component	Number
d – Decayed teeth	______
m – Missing teeth due to caries	______
f – Filled teeth	______
Total dmft	______
Permanent Dentition (DMFT)	
Component	Number
D – Decayed teeth	______
M – Missing teeth due to caries	______
F – Filled teeth	______
Total DMFT	______
Caries Status	
Variable	Result
Caries Present	☐ Yes ☐ No
dmft/DMFT Score	__________
